# High-throughput parallel testing of ten photoelectrochemical cells for water splitting: case study on the effects of temperature in hematite photoanodes†

**DOI:** 10.1039/d4se00451e

**Published:** 2024-05-30

**Authors:** Roberto Valenza, Isaac Holmes-Gentle, Franky E. Bedoya-Lora, Sophia Haussener

**Affiliations:** a Laboratory of Renewable Energy Science and Engineering, Institute of Mechanical Engineering, École Polytechnique Fédérale de Lausanne 1015 Lausanne Switzerland sophia.haussener@epfl.ch

## Abstract

High-throughput testing of photoelectrochemical cells and materials under well-defined operating conditions can accelerate the discovery of new semiconducting materials, the characterization of the phenomena occurring at the semiconductor–electrolyte interface, or the understanding of the coupled multi-physics transport phenomena of a complete working cell. However, there have been few high-throughput systems capable of dealing with complete cells and applying variations in real-life operating conditions, like temperature or irradiance. Understanding the effects of the variations of these real-life operating conditions on the performance of photoelectrode materials requires reliable and reproducible measurements. In this work, we report on a setup that simultaneously tests ten individual, identical photoelectrochemical cells whilst controlling temperature. The effects of temperature from 26 to 65 °C were studied in tin-doped hematite photoanodes for water splitting – as a reference case – through cyclic voltammetry and electrochemical impedance spectroscopy. The increase of surface-state-mediated charge recombination with temperature mainly penalized the energy conversion efficiency due to the reduction of the photovoltage produced. For parallel measurements in the ten individual cells, standard deviations from 20 to 60 mV for the onset potentials and less than 0.2 mA cm^−2^ for saturation current densities quantified the reproducibility of the results.

## Abstract 2

A setup that simultaneously tests ten photoelectrochemical cells whilst controlling temperature was developed. Applied to Sn:α-Fe_2_O_3_ photoanodes, increasing temperature penalized energy conversion efficiency due to increased surface recombination.

## Graphical abstract

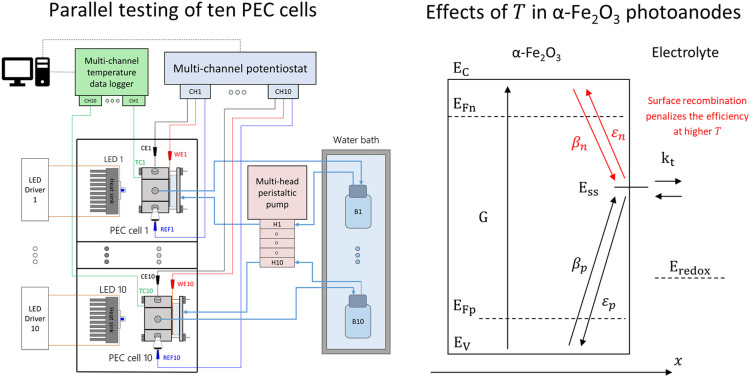

## 1 Introduction

Approaches for the photoelectrochemical (PEC) production of fuels, *i.e.* that convert molecules like H_2_O or CO_2_ into value-added chemicals (*e.g.* H_2_, CO, CH_4_ or more complex hydrocarbons) using at least one semiconductor–electrolyte junction irradiated by sunlight, represent a promising route for sustainable energy conversion and storage.^1^ PEC experiments are commonly performed in a single cell and reproducibility is not always assessed and quantified. However, reproducibility and uncertainty are important factors when considering the credibility of experimental results of different semiconducting materials, when studying their degradation, or when assessing how cell design choices affect performance.^2,3^ High-throughput systems with rapid serial or highly parallelized measurements can increase the data acquisition rate, allowing for a faster quantification of reproducibility.^4,5^ They can also accelerate the characterization of phenomena requiring long time scales and with a stochastic nature, *e.g.* the photocorrosion of a semiconductor after the formation of a pinhole in a protective layer.^6^ Various high-throughput systems for preparation and testing of semiconducting materials for PEC applications have been developed (Table S1†). The potential of automation in synthesis and characterization techniques for solar fuels production to accelerate the implementation and deployment of PEC technologies has also been recently highlighted.^7^ Researchers have focused on the high-throughput optimization of a number of key variables such as semiconductors composition,^8–17^ dopant materials and their concentration,^18–21^ structure directing agents,^22^ co-catalyst composition^23,24^ and deposition time.^25^ These high-throughput investigations typically focus on the PEC materials with a single measurement cell (or robotic arm) passing across the various material samples for their characterization. There are no examples of high-throughput PEC systems which reproduce changes in real-life operating conditions, *e.g.* irradiance or temperature, while performing multiple parallel and independent experiments. This strategy however has been successfully used to characterize the performance of batteries^26,27^ and membrane-electrode assemblies,^28,29^ both at the material as well as at the cell scale. Here, we took inspiration from these fields to develop a series of individual PEC cells that can be independently characterized. Their temperature or irradiation conditions can be separately controlled in order to collect data on the temperature-dependent characteristics of these semiconducting materials or PEC cells.

**Fig. 1:**
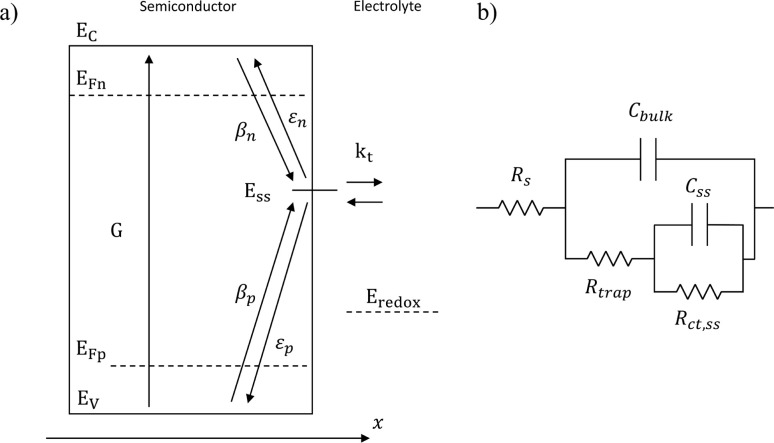
(a) Graphical representation of the simplified physical model for surface states-mediated charge transfer and recombination at a semiconductor–electrolyte interface proposed by Bertoluzzi and Bisquert.^42^ (b) Equivalent circuit describing the proposed physical model at the semiconductor–electrolyte interface:^41^*C*_bulk_ is the series connection of the Helmholtz double layer capacitance *C*_H_ and the space charge capacitance *C*_SC_; *C*_ss_ is the surface-state capacitance, being a chemical capacitance;^57,58^*R*_s_ is the series resistance; *R*_trap_ is the electrons trapping/detrapping resistance; and *R*_ct,ss_ is the resistance of charge transfer to/from surface states.

Variations in the operating temperature of PEC devices can be associated to the geographic location, the fluctuations of ambient temperature, or the intensity of the solar irradiation. The latter is especially relevant when concentrated radiation is used in the absence of appropriate thermal management.^30^ In the photovoltaic (PV) research community, the role of temperature has been accurately investigated and the concept of temperature coefficients has been introduced.^31–36^ These studies show that the efficiency of PV cells at the maximum power point decreases with temperature, mainly due to a decrease in the open-circuit voltage caused by larger recombination rates. A semiconductor–electrolyte interface may not be adequately modelled by a PV cell coupled with an electrochemical reaction at its surface.^37^ Moreover, semiconductor–electrolyte interfaces typically form surface states,^38–40^ which in metal oxide semiconductors for water splitting were proposed to be one (or more) surface hydroxyl (M–OH_*x*_) intermediate state(s) formed during the electrochemical reaction.^41^ Bertoluzzi and Bisquert^42^ described the competition between surface states-mediated charge transfer and recombination with a simplified analytical model as schematically shown in Fig. 1(a). This model produces an impedance which can be described with the equivalent circuit of Fig. 1(b), as proposed by Klahr *et al.*^41^ The mechanisms by which the temperature affects light absorption, charge separation, recombination and transfer, or photoelectrode stability in PEC devices is still under debate.^43–51^ There are no published studies for PEC water splitting focusing on how the competition between surface-state-mediated recombination and charge transfer is affected by temperature. Although there are a few studies on interfacial recombination in dye-sensitized solar cells, which report a decrease in performance as a result of increasing temperature.^52,53^

In this work, a setup to simultaneously test ten PEC cells while controlling temperature is presented. As a reference case, thin films of tin-doped hematite (Sn:α-Fe_2_O_3_) prepared by spray pyrolysis were tested with this setup at different temperatures to quantify the reproducibility of the parallel experiments. α-Fe_2_O_3_ was chosen due to its earth abundance, non-toxicity, and stability in alkaline environment,^54^ despite its extremely small charge lifetime (in the order of 10 ps (ref. 55)) and poor minority charge mobility (0.2 cm^2^ V^−1^ s^−1^ (ref. 56)), leading to a hole diffusion length of only 2–4 nm. The effect of temperature from 26 to 65 °C on light absorption, charge separation, recombination and transfer in the semiconductor–electrolyte junction was evaluated by cyclic voltammetry (CV) and electrochemical impedance spectroscopy (EIS).

## 2 Experimental

### 2.1 Hematite photoanode preparation

Sn-doped α-Fe_2_O_3_ thin films were deposited on conductive glass *via* spray pyrolysis following a previously reported procedure.^59^ Briefly, 0.1 M FeCl_3_·6H_2_O (>99%, Acros Organics) and 0.6 mM SnCl_4_ (anhydrous, 99%, Thermo Fisher Scientific) were dissolved in absolute ethanol (99.8% Fisher Chemical). Sn^4+^ concentration corresponds to *ca.* 1.3% doping by mass. The precursor was nebulized with a quartz spray nozzle (Meinhard, USA) at a height of 150 mm above the surface of the substrate, which was kept at 450 °C. 20 passes of precursor flowing at 2 cm^3^ s^−1^ were sprayed onto semitransparent fluorine-doped tin oxide (FTO) coated glass (Solaronix TC22-15, 2 mm). A 60 s rest between passes was necessary to allow the precursor to completely evaporate the solvent. The samples were then annealed at 400 °C in air for 1 h. Electrical contact was made by attaching copper conductive tape (contact resistivity 4.7 × 10^−3^ Ω m, 3 M) on bare FTO at the top of the samples.

### 2.2 UV-visible spectroscopy and scanning electron microscopy

Reflectance and transmittance spectra of the Sn:α-Fe_2_O_3_ photoelectrodes were measured with a Shimadzu UV-2600 UV-vis-NIR spectrophotometer with an integrating sphere (ISR-2600 PLUS Shimadzu). Absorbance spectra were then calculated and the Tauc equation was fitted, assuming a direct optical transition, to obtain the optical energy bandgap of the semiconductor at ambient temperature. Scanning electron microscopy (SEM) images of the surface and the cross section of the Sn:α-Fe_2_O_3_ photoelectrodes were taken with a Field Emission SEM (Zeiss Merlin). See ESI Note 1 for detailed information.†

### 2.3 PEC test cell design

Parallel electrochemical tests were performed in a three-electrode configuration: coiled platinum wires were used as counter electrodes and Gaskatel Hydroflex reversible hydrogen electrodes (RHE) as reference electrodes. The circular coiled shape of the counter electrode, which was placed as a continuous ring around the working electrode, reduces the current density distribution over the sample. The electrolyte temperature inside the cells next to the photoelectrodes was measured by K-type thermocouples connected to two Pico Technology TC-08 data loggers. The body of the cell is made from chemically stable polyether ether ketone (PEEK), for the parts in contact with the alkaline electrolyte, and polyoxymethylene (POM) for the rest of the components. A quartz window (Knight Optical, transmittance of 94% in the wavelength range 400–500 nm, thickness 3 mm) and ethylene propylene diene monomer (EPDM) O-rings were used. Silicone gaskets with a circular hole of 8 mm defined the photoelectrode geometrical active area of 0.5 cm^2^. The exploded-view technical drawing and annotated photograph of the PEC cell are reported in Fig. S5.†

### 2.4 Setup of array of ten PEC cells

**Fig. 2:**
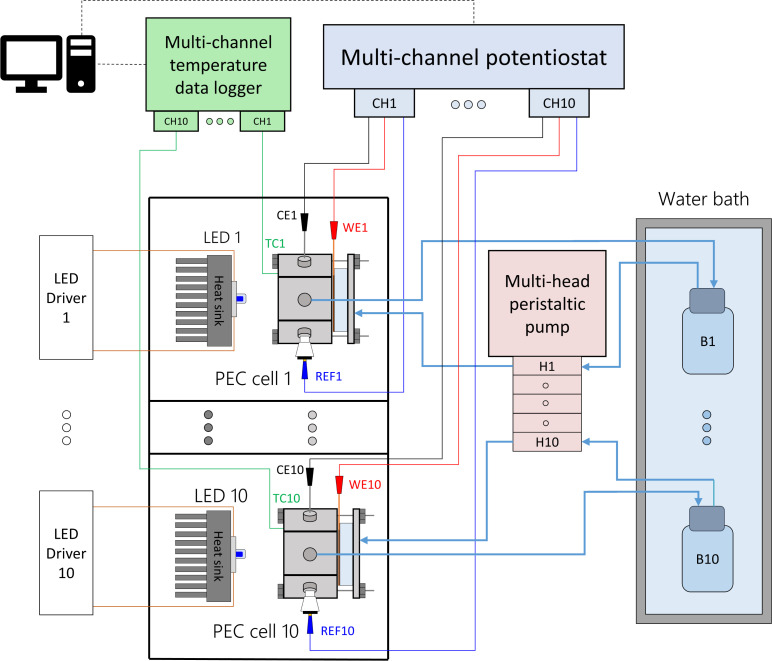
Simplified schematics of the modular experimental setup to test ten PEC cells in parallel at different temperatures.

Each PEC cell had an independent hydraulic circuit driven by one of two multi-channel Shenchen LabV1 peristaltic pumps with MC12 pump heads. The electrolyte reservoirs, one for each cell, were glass bottles placed inside a 28 L water bath (Fisherbrand Isotemp). Parallel experiments were performed by connecting the cells to a ten-channel potentiostat/galvanostat (MultiPalmSens4). The light sources were blue LEDs (peak wavelength 442 nm, ILS OSLON SSL4). The LEDs were powered by constant-current LED drivers (0.7 A, ILS IZC070) and attached to aluminium heat sinks by thermal adhesive. The distance from the LED to the semiconductor surface was fixed to 81 mm, corresponding to an irradiance of 384 W m^−2^. This value was chosen to simulate the useful irradiation that could be harvested from the global standard spectrum (AM 1.5G) by the hematite photoanodes. Detailed calculations can be found in ESI Note 2.† Each light source and PEC cell was operated in an independent optical enclosure constructed from black hardboard (Thorlabs) and aluminum profiles (Bosch). A simplified schematic and photos of the developed setup are shown in Fig. 2, S6 and S7,† respectively.

### 2.5 PEC measurements

The performance of Sn-doped α-Fe_2_O_3_ photoelectrodes was evaluated by CV and EIS in dark and under front illumination by a blue LED, *i.e.* light passes sequentially through the 1 M NaOH aqueous electrolyte solution (pH ≈ 13.6, electrolyte layer thickness 1.7 cm) before reaching the electrolyte–semiconductor interface. Mean temperatures of the temporal averages of ten parallel experiments were fixed to 26, 36, 44, 56 and 65 °C. The measured temperatures of the ten cells are reported in Fig. S8.† All error bars reported in this manuscript are obtained from the standard deviation of the ten parallel experiments. Fitted curves of the experimental data are reported in dashed lines.

#### 2.5.1 Cyclic voltammetry

CVs were performed at a scan rate of 20 mV s^−1^ from 0.6 to 1.9 V *vs.* RHE. The average of the third forward sweep of each of the ten parallel measurements is presented. Before each measurement, the cell was left at the set temperature for approximately 20 min to reach steady state. In this work, and only for comparison purposes, the onset potential is defined as the electrode potential at 0.2 mA cm^−2^, in a similar way as suggested by Huang *et al.*^45^ The saturation current density (*j*_sat_) is defined as the current density at 1.5 V *vs.* RHE. Photocurrent density (*j*_ph_) is defined as the difference between the current density under illumination and in dark at a given photovoltage value (eqn (1)). The photovoltage (*V*_ph_) is defined as the difference in the potential between dark and light conditions at a given photocurrent value (eqn (2)).^60^

1 *j*_ph_ = *j*_light_|_*V*_ph__ − *j*_dark_|_*V*_ph__

2 *V*_ph_ = *E*_dark_|_*j*_ph__ − *E*_light_|_*j*_ph__

Apparent short-circuit photocurrents (*j*_sc_) were calculated by linear fitting of the saturation region of the photocurrent–photovoltage characteristic curves as proposed by Huang *et al.*^45^ The onset photovoltage (*V*_ph,on_) is defined as the photovoltage at which a photocurrent of 0.2 mA cm^−2^ is measured.

#### 2.5.2 Energy efficiency calculation

The applied bias photon-to-current efficiency (ABPE) is calculated considering the temperature dependence of the equilibrium potential and assuming a unitary oxygen activity (eqn (3)).^61^ As a performance metric, ABPE has been found to have a number of issues when assessing photoelectrochemical devices under electrical bias.^62,63^ Nevertheless, ABPE can be a useful tool for comparing the performance of a given material subjected to different operating conditions, although care must be taken as ABPE values should not be compared with other reports for different materials or devices.3
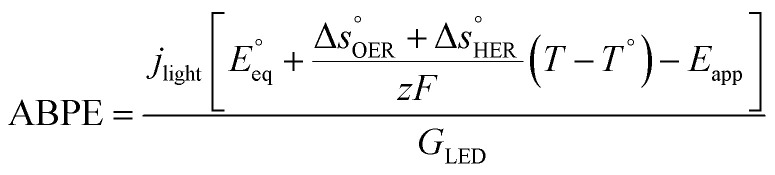

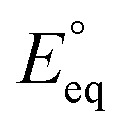
 is the standard equilibrium potential of the oxygen evolution reaction (OER) at 25 °C (1.23 V *vs.* RHE), 
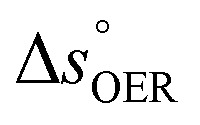
 is the standard entropy of the OER, 
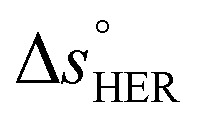
 is the standard entropy of the hydrogen evolution reaction (HER) to account for the shift of the hydrogen reference electrode with temperature, *z* is the number of moles of electrons transferred in the half reaction per moles of reactant, *F* is the Faraday constant 
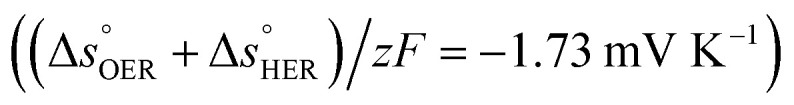
,^64,65^*T* the average cell temperature, *T*° is the standard temperature (25 °C), *E*_app_ is the applied potential, *G*_LED_ is the total average irradiance from the blue LED (384 W m^−2^). The apparent photon conversion efficiency (PCE) is obtained as the product of photocurrent and photovoltage over the LED total irradiance (eqn (4)).4
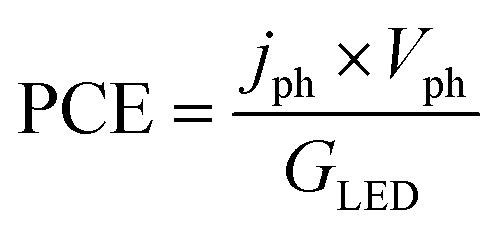


#### 2.5.3 EIS measurements

EIS was performed by applying potentials from 0.8 to 1.5 V *vs.* RHE with a sinusoidal amplitude of 20 mV and frequencies from 100 kHz to 0.1 Hz. The Nyquist and Bode plots of each measurement under light were fitted using the equivalent circuit proposed by Klahr *et al.*^41^ (Fig. 1(b)). A Matlab script (Zfit^66^) was used to fit the experimental data to the equivalent circuit using a tolerance of 10^−8^ Ω cm^2^ for the optimization function. From the fitted curves of the ten parallel measurements, the average resistances and capacitances and their standard deviations were calculated. Dark EIS spectra were fitted using a Randles circuit (*R*(*RC*)). The flat band potential (*E*_fb_) and the concentration of ionized donors (*N*^+^_D_) were estimated with eqn (5) from the Mott–Schottky plots assuming a planar one-dimensional photoelectrode thicker than the depletion region. The depletion layer thickness was calculated assuming uniform donor distribution with eqn (6).^67^5
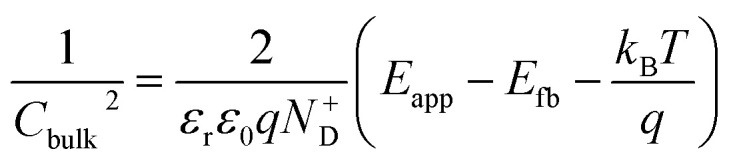
6
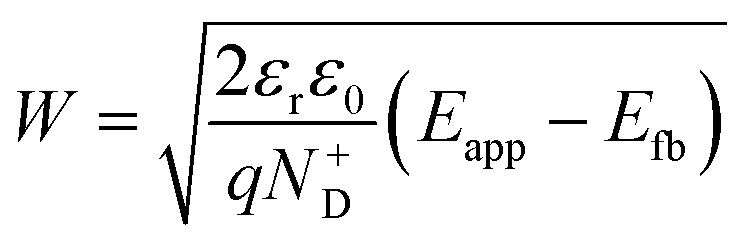
*ε*_r_ is the relative permittivity of Sn:α-Fe_2_O_3_, assumed to be equal to 32 and temperature-independent between 26 °C to 65 °C,^39,68^ and *ε*_0_ is the vacuum permittivity. The density of surface states (DOS) was calculated from the surface state capacitance *C*_ss_ (eqn (7)) and the total charge of surface states (*Q*_tot_) was obtained fitting *C*_ss_ with a Gaussian function and integrating the latter over the potential (eqn (8)).^41^7
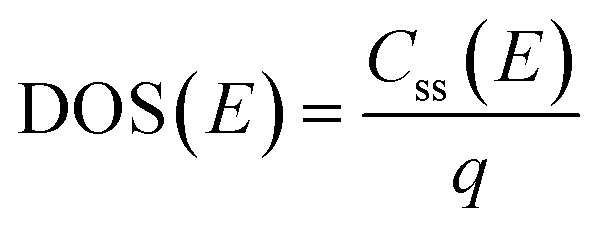
8
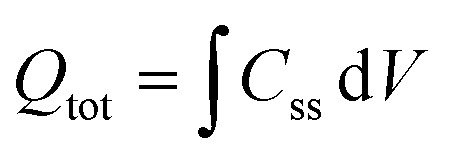


From the analytical derivation proposed by Bertoluzzi *et al.*,^42^ the ratio between the electron trapping–detrapping kinetic constants (*ε*_n_ + *β*_n_*n*) and the charge transfer from surface states kinetic constant (*k*_t_) is calculated with eqn (9). As proposed by Wijayantha *et al.*,^69^ the charge transfer efficiency was obtained from the fitted *R*_trap_ and *R*_ct,ss_*via*eqn (10).9
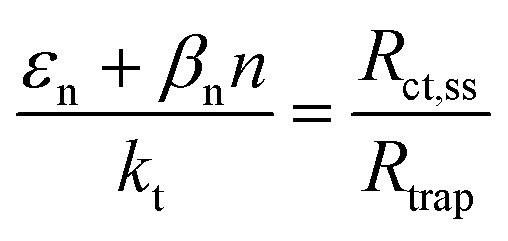
10
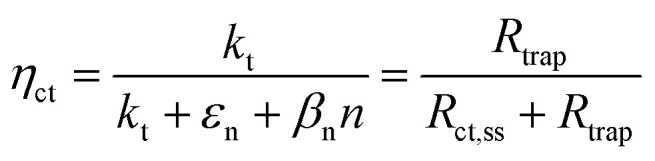


## 3 Results and discussion

### 3.1 Capacitive response of the Sn:α-Fe_2_O_3_–electrolyte junction

**Fig. 3:**
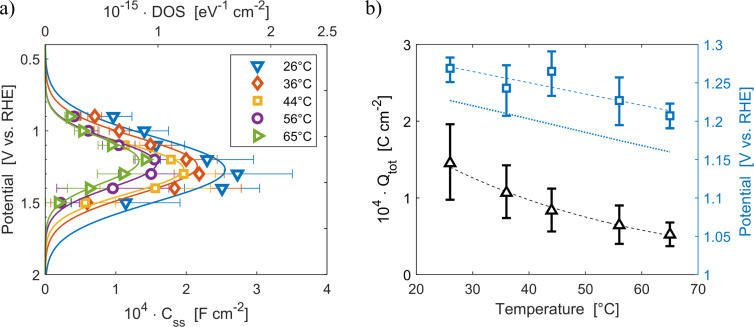
(a) Density of surface states (top axis) and surface states capacitance (bottom axis) of Sn:α-Fe_2_O_3_ thin films as a function of applied potential at different temperatures. (b) Total surface states charge fitted with the exponential function of eqn (11) (black triangles, left axis) and surface states energy level (blue squares, right axis) compared to the OER equilibrium potential assuming unitary activity of oxygen accounting for the hydrogen reference electrode shift in potential due to temperature (blue dotted line, right axis), all as a function of temperature. The dashed lines are the regressions of the data.

The density of surface states, derived from the surface states capacitance with eqn (7), can be described by a Gaussian function with respect to the applied potential at all the five analyzed temperatures from 26 °C to 65 °C (Fig. 3(a)). As observable in Fig. 3(b), the total charge of surface states decreased from 1.4 × 10^−4^ C cm^−2^ at 26 °C to 0.5 × 10^−4^ C cm^−2^ at 65 °C. This behavior was fitted to an exponential function of temperature, as expressed in eqn (11).11
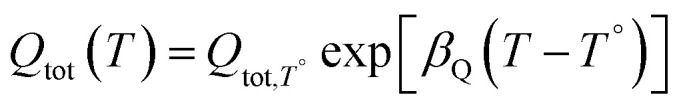
*β*_Q_ is a temperature coefficient found to be −2.6 × 10^−2^ K^−1^. The energy level of the surface states, *i.e.* the peak of the Gaussian fitting function, decreases from 1.27 to 1.21 V *vs.* RHE when increasing the temperature from 26 to 65 °C, with an average rate of −1.5 mV K^−1^ (Fig. 3(b), right axis). The comparable decrease of the equilibrium potential of OER with temperature accounting for the shift of the hydrogen reference electrode (−1.73 mV K^−1^)^64,65^ supports the previously reported hypothesis that the surface states follow a Nernstian behavior.^41^

**Fig. 4:**
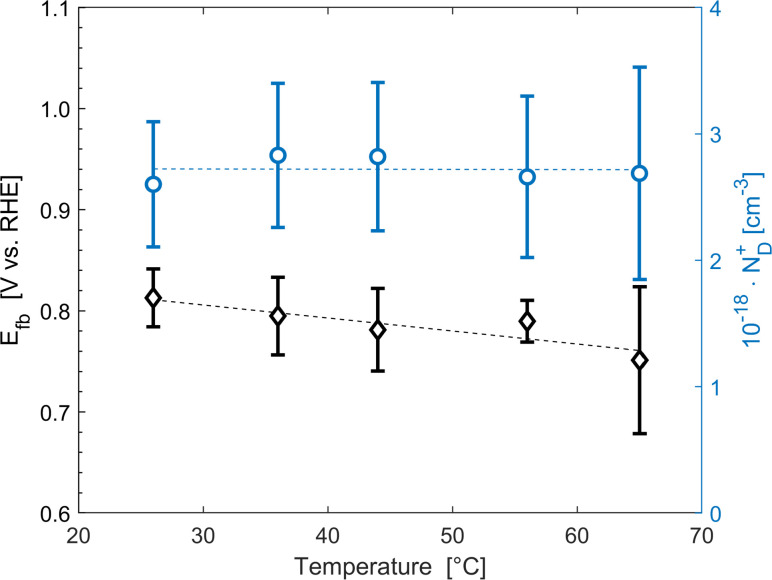
Flat band potential (black diamonds, left axis) and concentration of ionized donor density (blue circles, right axis) as a function of temperature. The dashed lines are the regressions of the data.

The average concentration of ionized donors, calculated from the Mott–Schottky plots (in the dark) shown in Fig. S9,† was found to be constant with temperature and equal to 2.7 × 10^18^ cm^−3^ (Fig. 4), in agreement with the value of 4.5 × 10^18^ cm^−3^ previously reported in literature for the same material.^59^ In the temperature range under examination, it is a reasonable assumption that all the dopant atoms in the lattice are ionized^70^ and temperature does not affect donor concentration. In previous studies, donor concentration was found to increase with temperature due to possible thermally-activated donor sites^45,71^ but this was not observed for this doping level. The flat band potential shifted from 0.81 V *vs.* RHE at 26 °C to lower potentials at a rate of −1.3 mV K^−1^. This variation could be caused by possible temperature-induced modifications of Sn:α-Fe_2_O_3_ surface dipoles at the interface with the electrolyte inducing a band shift^72,73^ and by the narrowing of the semiconductor bandgap *E*_g_ with temperature according to Varshni model^74^ (eqn (12)).12
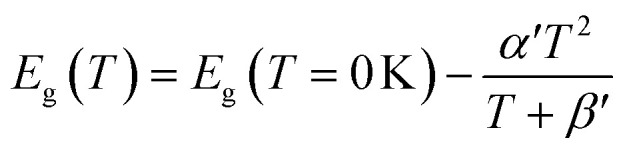
*α*′ is the limit of the bandgap entropy when *T* approaches positive infinity and *β*′ is expected to be comparable with the Debye temperature of a given material. For Ti-doped hematite photoelectrodes, the energy bandgap variation with temperatures from 20 to 66 °C has been estimated to be −1.2 meV K^−1^.^45^ Moreover, the flat band potential shift can be caused by the change in the energy difference between the conduction band edge *E*_C_ and the Fermi level of electrons *E*_F_:13
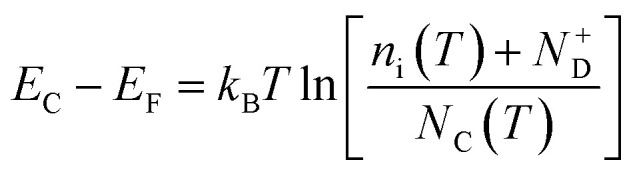
*k*_B_ is the Boltzmann constant, *n*_i_ is the semiconductor intrinsic carrier concentration and *N*_C_ the effective density of states of electrons in the conduction band. Apart from the explicit dependence, temperature also affects *n*_i_ and *N*_C_ as:14
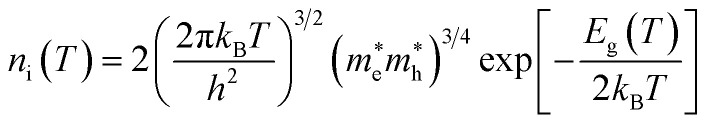
15
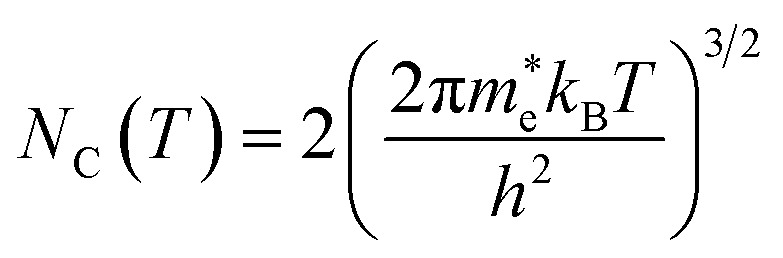
*h* is the Planck constant, 
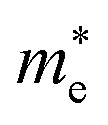
 and 
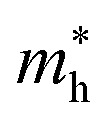
 are the effective masses of electrons and holes, which are known to be temperature dependent in most semiconducting materials, although for the analyzed temperature range they could be considered constant.^75–77^ Physics-based modelling of the semiconductor–electrolyte interface^78,79^ can decouple the contribution of these different effects, but it is out of the scope of the present work.

**Fig. 5:**
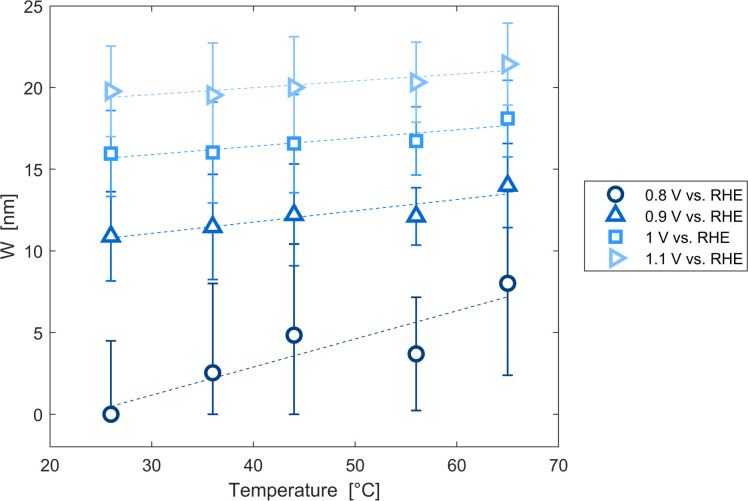
Space charge layer depth assuming a planar semiconductor thicker than the depletion layer as a function of temperature for applied potentials from 0.8 to 1.1 V *vs.* RHE and linear regressions of the data (dashed lines).

The decrease of the flat band potential, under the assumptions of a planar semiconductor thicker than the depletion layer, implies an increase of space charge layer depth at fixed applied potential (Fig. 5). The effect is more pronounced at potentials close to the flat band potential: the average rate is 0.17 nm K^−1^ at 0.8 V *vs.* RHE and it decreases to 0.04 nm K^−1^ at 1.1 V *vs.* RHE. Separation of charge carriers through migration is therefore favoured by temperature and band bending is more pronounced at constant applied potentials close to the flat band condition (for a temperature-independent donor concentration). The Mott–Schottky plots of the Sn:α-Fe_2_O_3_ thin films under blue light at different temperatures show the deviation from the linear trend of the same curves in dark due to surface state pinning^39,41^ (Fig. S10†).

### 3.2 Cyclic voltammetry and energy efficiency calculations

**Fig. 6:**
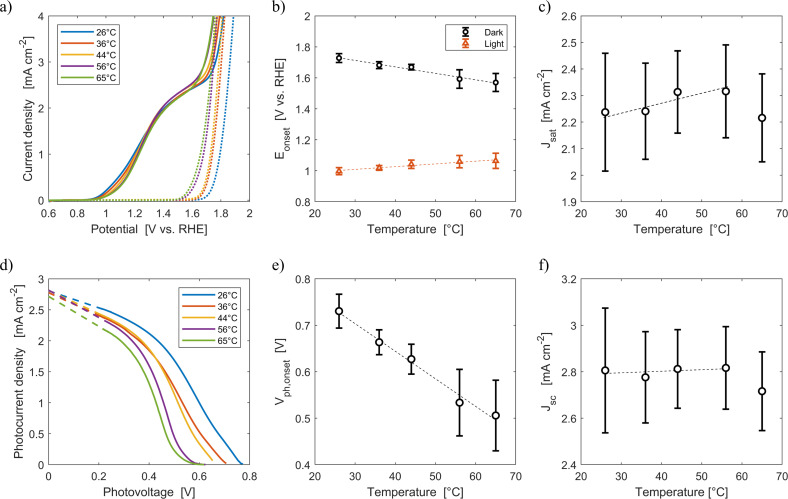
(a) Current density-applied potential characteristic curves of Sn:α-Fe_2_O_3_ thin films at different temperatures under blue (*ca.* 440 nm) light (solid lines) and in dark (dotted lines). (b) Onset potential as a function of temperature in dark and light. (c) Saturation current density as a function of temperature. (d) Photocurrent–photovoltage characteristic curves of Sn:α-Fe_2_O_3_ thin films at different temperatures and linear regressions of the saturation region of the curves (dashed lines). (e) Onset photovoltage as a function of temperature. (f) Apparent short-circuit photocurrent as a function of temperature. In (b), (c), (e) and (f) the dashed lines are the linear regressions of the experimental data.

Fig. 6(a) shows the average current densities measured during the third forward sweep of each of the ten parallel cyclic voltammetries with Sn:α-Fe_2_O_3_ at five different average temperatures (26 °C, 36 °C, 44 °C, 56 °C, 65 °C) under blue light and in dark. The curves with error bars can be found in Fig. S11.†Fig. 6(d) shows the corresponding photocurrent density as a function of the photovoltage. The same curves with error bars are reported in Fig. S12.† As highlighted in Fig. 6(b), the onset potential of the reaction in dark decreases from 1.73 V *vs.* RHE at 26 °C to 1.57 V *vs.* RHE at 65 °C with an average rate of −4.2 mV K^−1^. This reduction follows the shift downwards of the OER equilibrium potential, but more importantly it is caused by the improved kinetic rate constant of the electrochemical reaction.^43,46^

Under illumination, the decrease of the total charge of surface states and the shift of their energy level towards lower potentials, as observed *via* EIS in Fig. 3(a) and (b), cannot be directly observed by CV. Surface states indeed have two competing roles as active centers for the indirect charge transfer and as surface recombination centers. The holes and electrons surface quasi-Fermi level difference decreases with temperature (Fig. 6(e)). The onset potential under illumination increases from 1.00 V *vs.* RHE at 26 °C to 1.06 V *vs.* RHE at 65 °C at a rate of +1.8 mV K^−1^ (Fig. 6(b)). The same trends have already been observed in previous reports.^45,46^ This is caused by a lower surface hole density at higher temperatures mainly due to a more significant increase in recombination compared to the less significant improvements in reaction kinetics and in charge separation.

The saturation current density increases from 2.24 mA cm^−2^ at 26 °C to 2.32 mA cm^−2^ at 56 °C with a rate of 3 μA cm^−2^ K^−1^. These values are higher than those previously reported for the same material,^59^ likely due to a better spectral efficiency of hematite under blue illumination (*ca.* 440 nm) with respect to the average one when illuminated by the AM 1.5G spectrum. However, these values at fixed applied potential are affected by a variation of the internal photovoltage and by light management in the material. Therefore, the apparent short-circuit current of the material is extracted to isolate the temperature effects on light management. The results show that *J*_sc_ can be considered temperature-independent up to 56 °C (rate of +0.7 μA cm^−2^ K^−1^, with a relative rate lower than 0.025% K^−1^). This is because the narrowing of the bandgap with temperature (eqn (12)) does not change the number of absorbed photons from the monochromatic blue LED (it rather increases the thermalization losses), and the absorption coefficient of hematite should not be affected by temperature in the wavelength range emitted by the LED.^45^ The spectral response of the material at photovoltages equal to zero for wavelengths between 400 and 500 nm can therefore be considered not affected by temperature. The reduction of saturation and apparent short-circuit currents at 65 °C can potentially be associated to the instability of the material.^43^ Huang *et al.* observed a similar behaviour for temperatures higher than 60 °C in Ti-doped α-Fe_2_O_3_ photoelectrodes. The reduction of apparent short-circuit current was measured despite the predicted larger number of absorbed photons due to the decrease of the material bandgap with temperature and the use of simulated AM 1.5G spectrum.^45^ Additional research possibly using SEM and inductively coupled plasma mass spectroscopy^80^ is required to better understand this behavior.

ABPE of the Sn:α-Fe_2_O_3_ thin films decreased with temperature at any applied potential mainly due to the more pronounced surface recombination with temperature, which lowers the current at potentials lower than 1.2 V *vs.* RHE (Fig. S13(a)†). The peak of ABPE linearly decreases with temperature from 0.18% at 26 °C to 0.05% at 65 °C (Fig. S13(b)†). PCE has a decreasing trend with temperature for photovoltages greater than 0.2 V, where surface recombination has a more dominant effect (Fig. S13(c)†). The peak of PCE linearly decreases with temperature from 2.3% at 26 °C to 1.6% at 65 °C with a similar trend to its photovoltage (Fig. S13(d)†).

### 3.3 Resistive response of the Sn:α-Fe_2_O_3_–electrolyte junction

**Fig. 7:**
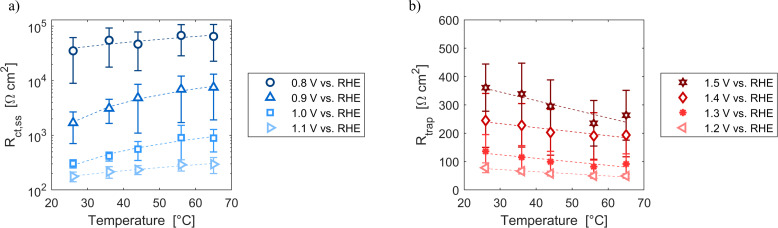
(a) Charge transfer resistance from the surface states at potentials from 0.8 to 1.1 V *vs.* RHE as a function of temperature. (b) Trapping–detrapping resistance at potentials from 1.2 to 1.5 V *vs.* RHE as a function of temperature. In (a) and (b) the dashed lines are the linear regressions of the data.

Resistances obtained fitting the EIS spectra are associated to the steady-state response of the semiconductor–electrolyte junction. The operation of the system is characterized by two distinct regions: for potentials lower than 1.2 V *vs.* RHE, the charge transfer resistance from surface states is the most significant contribution to the total resistance of the system; for potentials higher or equal than 1.2 V *vs.* RHE, the trapping–detrapping resistance of electrons is the dominant one (Fig. S14†). The trend with temperature of *R*_tot_ is strongly related to the ones of *R*_ct,ss_ and *R*_trap_ in the two operating regimes: *R*_tot_ increased for potentials lower than 1.2 V *vs.* RHE and it decreased for higher potential values (Fig. 7). For instance, *R*_tot_ linearly increased with temperature with an average rate of 744 and 159 Ω cm^2^ K^−1^ at 0.8 and 0.9 V *vs.* RHE, respectively, and *R*_ct,ss_ increased at a rate of 743 and 158 Ω cm^2^ K^−1^ at the same potentials. At 1.4 and 1.5 V *vs.* RHE, *R*_tot_ decreased at rates of −1.2 and −2.9 Ω cm^2^ K^−1^, respectively, which are similar to the rates of −1.3 and −3.0 Ω cm^2^ K^−1^ of *R*_trap_ at the same potentials. A minimum of *R*_ct,ss_ and *R*_tot_ was observed at the surface state energy level, the peak of the Gaussian fitting function of Fig. 3(b). It supports the hypothesis that the charge transfer is mediated by surface states.^41^

**Fig. 8:**
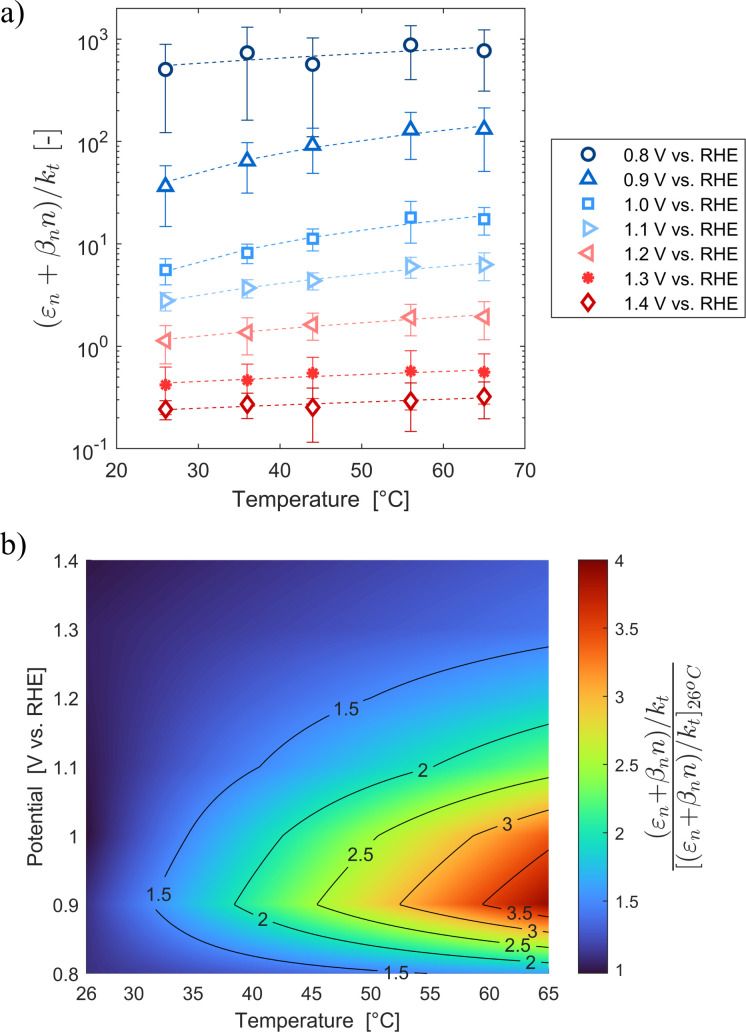
(a) Ratio between electron trapping–detrapping kinetic constants and charge transfer from surface states kinetic constant of the Sn:α-Fe_2_O_3_ thin films at potentials from 0.8 to 1.4 V *vs.* RHE as a function of temperature and (b) the same ratio normalized with its value at 26 °C linearly interpolating data at potentials from 0.8 to 1.4 V *vs.* RHE as a function of temperature.

**Fig. 9:**
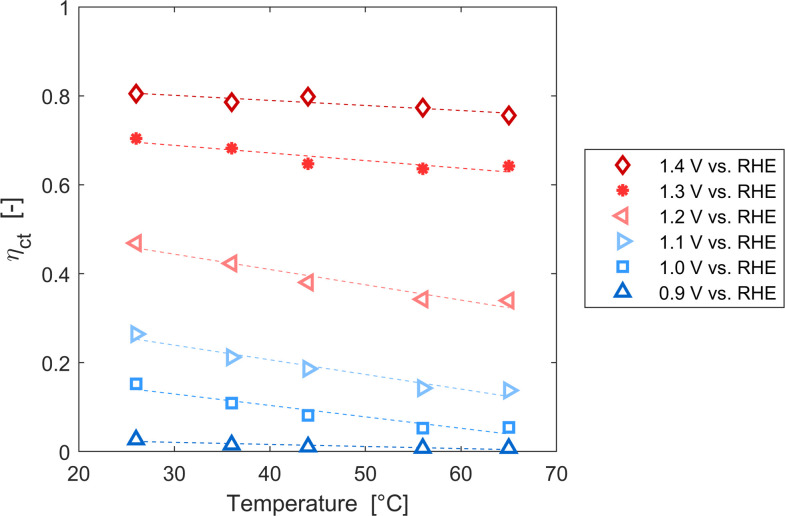
Charge transfer efficiency of the Sn:α-Fe_2_O_3_ thin films at potentials from 0.9 to 1.4 V *vs.* RHE as a function of temperature.

These variations are caused by a larger relative increase of the kinetic constants of charge recombination (*ε*_n_ + *β*_n_*n*) compared to the kinetic constant of charge transfer from surface states (*k*_t_) with temperature, as observed in Fig. 8(a). This ratio decreases by almost four orders of magnitude from 0.8 to 1.4 V *vs.* RHE due to the less significant surface recombination at higher potentials. An increase with temperature is observed at every potential. Normalizing the ratio (*ε*_n_ + *β*_n_*n*)/*k*_t_ with its value at 26 °C, it can be noted how the most significant increase occurs between 0.9 and 1.0 V *vs.* RHE, where a factor 4 is almost reached at 65 °C (Fig. 8(b)). These variations also imply that the charge transfer efficiency decreases with temperature at every potential, especially between 1.0 and 1.3 V *vs.* RHE where an average rate of −0.3% K^−1^ was observed (Fig. 9).

The series resistance, associated to the ohmic phenomena in the semiconductor, the electrolyte and the conductive substrate, is not affected by the applied potential and is the smallest in magnitude (Fig. S14(c)†). The conduction of electrons in transition metal oxides like α-Fe_2_O_3_ is governed by small polaron hopping.^81,82^ Small polarons are electrons that are self-trapped in a single-site local lattice distortion, which for Ti-doped hematite is predicted to be nearby a Fe^3+^ ion, reducing it to Fe^2+^.^83^ To hop from one site to the next, small polarons require a phonon and the process has an activation energy which follows an Arrhenius-type temperature dependence (under the assumption of adiabatic regime). The increase in the semiconductor conductivity with temperature has been proved to increase the minority carrier diffusion length in metal oxide semiconductors.^48,49^ Zhang *et al.*^48^ reported an increase of current density with temperature at 1.23 V *vs.* RHE for Ti:α-Fe_2_O_3_ thin films at a rate 0.6% K^−1^ but they used a hole scavenger to suppress the effects of surface recombination and a solar simulator as light source. Therefore, their values cannot be directly compared with the observed increase of saturation current density reported in Fig. 6(c). At low applied potentials, when the space charge layer depth is small, this effect positively contributes to an increase in the amount of charge which can be collected in the quasi neutral region of the material. However, an increase in current could not be observed due to the larger increase of surface recombination rates. The conductivity of the electrolyte increases with temperature due to the higher degree of ion dissociation, producing a larger number of free ions, and the reduction of the intramolecular forces between ions, which increases the mobility of charges.^84,85^ However, as shown by Dias *et al.*,^44^ the main contribution to the series resistance is given by the FTO conductive layer. Although it has been observed that the electron mobility in this material decreases with temperature,^86^ the reduction of the series resistance in metal oxide semiconductor–electrolyte junctions was previously observed,^43,45^ indicating that further research about ohmic phenomena in these systems is required.

### 3.4 Reproducibility of results in the array of ten PEC cells

The effects of temperature in Sn:α-Fe_2_O_3_ semiconductor–electrolyte junctions were analyzed from the averages of parallel experiments in the developed array of ten PEC cells. However, these measurements were characterized by uncertainties which will be discussed below to determine the reproducibility of the results, proposing strategies to improve it in future studies.

The onset potentials of Fig. 6(b) had an average standard deviation of 20 mV from 26 to 44 °C which increased to 50 mV at 56 and 65 °C. The variations of temperature among the cells are larger at higher temperature (Fig. S8†). The standard deviation of the measured temperature increased from 2.2 to 5.0 °C enhancing the average temperature from 26 to 65 °C. This effect was assumed to be the result of the more pronounced heat losses in the tubes of the cells placed further from the water bath (Fig. S6†). Using the temperature coefficients reported in Section 3.2, a variation in temperature of 7 °C, as the one observed at 65 °C between cells 2 and 5, would translate in a change of −29.4 mV and +12.6 mV of the onset potential in dark and light, respectively. To mitigate this effect, a thermal insulator at the outer surface of the polymeric tubes can be introduced. Hydrogen reference electrodes were characterized by a shift with respect to their theoretical value quantified by a standard deviation of approximately 5 mV. The accuracy of the potentiostat was less than 0.1% ± 0.1 mV of offset. Hence, the error associated to the potentiostat can be considered negligible towards the total variance observed in the measurements. Saturation current densities had an average standard deviation of 0.17 mA cm^−2^, almost constant with temperature (Fig. 6(c)). The average coefficient of variation of this quantity was 7.5%, which is in good agreement with the expected variation of the irradiance emitted by the LEDs (7.2%). This suggests that the LEDs were the main source of error in this measurement. The variations in the irradiance from the LEDs also affected the concentration of photogenerated carriers, introducing additional errors to the produced photovoltage. This variation in irradiance could be minimised by calibrating the optimal distance of each PEC cell from each LED, although this laborious task could introduce further errors related to accurate cell placement. A more superior but expensive solution would be the introduction of individual controllers for each LED allowing for minor adjustments in the applied current to reduce irradiance variability.

Resistances and capacitances were characterized by large standard deviations (Fig. S14†). Close to the flat band condition, the derivative of the *J*–*E* characteristic curve is almost zero. Small changes of the slope among different cells at the same potential generated large variations in the total resistance due to their inverse proportionality. Indeed, the coefficient of variation of *R*_tot_ averaged in temperature reached values above 60% for potentials lower than 1.0 V *vs.* RHE (Figure S15†(a)). The uncertainty in *R*_tot_ averaged in potential increased with temperature for the same reasons previously described for the onset potential and the photovoltage (Fig. S15(b)†). The use of galvanostatic EIS could decrease the errors in the resistances close to the flat band potential. Indeed, the slope of the characteristic curve at a fixed current is affected by smaller variations than at fixed potential. However, the evaluation of potential-dependent quantities like the energy level of surface states would not be directly accessible without an extrapolation.

## 4 Conclusions

In this work, a system to test ten PEC flow cells in parallel whilst controlling temperature was developed. The reproducibility in different cells of the system was quantified using Sn-doped α-Fe_2_O_3_ thin-films for PEC water splitting as reference case, studying the effects of temperature between 26 and 65 °C. Increasing temperature penalized the energy conversion efficiency of the material mainly due to a greater increase of surface recombination with respect to charge transfer from surface states. A decrease of the photovoltage produced by the material was observed, negatively impacting the charge transfer efficiency. Flat band potential shifted cathodically with increasing temperature, favouring charge separation through migration at fixed applied potentials. Due to the use of monochromatic LED light sources, it was not possible to assess the effect of the reduction of semiconductor bandgap with temperature; hence, the apparent short-circuit current did not increase with temperature. The series resistance decreased with temperature, although the effects of the improvement of minority carrier diffusion length due to the better small polaron hopping were not appreciable due to the larger increase of surface recombination rates.

Additional work is required to find strategies to selectively suppress surface recombination in semiconductor–electrolyte junctions at temperatures above environmental conditions to benefit from the observed improvements in electrochemical reaction kinetics, charge separation, and small polaron transfer. Deposition of passivating layers^87^ or catalysts like Co–Pi^88,89^ or IrO_*x*_ (ref. 90) at the surface of the photoelectrode could be possible solutions. The use of holes scavengers^91^ or of techniques like intensity modulated photocurrent or photovoltage spectroscopies^92,93^ and transient absorption spectroscopy^94,95^ could elucidate the role of surface states in the competition between charge transfer and recombination at different temperatures.

We presented a versatile setup with ten individual cells that can be exposed to varying operating conditions, and therefore be used not only to test materials in functional cell configurations but also complete working cells in order to assess how design or operation can affect the performance and longevity of PEC devices. This work is inspired by similar setups that have been developed in the battery or fuel cell community and may serve to increase the understanding of degradation and quantification of reproducibility, something that is still in its infancy in the PEC (device) community.

## Author contributions

Roberto Valenza: conceptualization, methodology, investigation, formal analysis, data curation, writing – original draft. Isaac Holmes-Gentle: conceptualization, methodology, writing – review & editing. Franky E. Bedoya-Lora: conceptualization, methodology, writing – review & editing. Sophia Haussener: funding acquisition, conceptualization, supervision, project administration, writing – review & editing.

## Conflicts of interest

There are no conflicts to declare.

## Supplementary Material

SE-008-D4SE00451E-s001
