# Supplementary material for: High-throughput parallel testing of ten photoelectrochemical cells for water splitting: case study on the effects of temperature in hematite photoanodes
Source: Sustain Energy Fuels. 2024 May 30;8(16):3583–94. doi: 10.1039/d4se00451e (PMC11302243; doi:10.1039/d4se00451e)
Supplement: SE-008-D4SE00451E-s001 [file SE-008-D4SE00451E-s001.pdf]

Electronic Supplementary Material (ESI) for Sustainable Energy & Fuels.
This journal is © The Royal Society of Chemistry 2024

Supporting Information - High-throughput parallel testing of ten photoelectrochemical cells for water splitting: case study on the effects of temperature in hematite photoanodes

Roberto Valenza, Isaac Holmes-Gentle, Franky E. Bedoya-Lora, Sophia Haussener

2024

Table S1: Literature review about high-throughput photoelectrochemical (PEC) setups.

Number of samples	PEC active area	Semiconductor(s)	Deposition method	Parallel experiments	Independent experiments	Flow of the electrolyte	Temperature control	Reference
342	$1.25 \cdot 10^{-3}$ cm^2	Ti-W-O	Magnetron sputtering	no	yes	yes	no	[1]
71	0.42 cm^2	Fe–Ti–W–O	Magnetron sputtering	no	yes	no	no	[2]
5608	1.2 $\cdot 10^{-2}$ cm^2	X-Sb-O (X: various elements)	Magnetron sputtering	no	yes	no	no	[3]
858	1 $\cdot 10^{-2}$ cm^2	$CuBi_2O_4$ with different oxides	Reactive co-sputtering	no	yes	no	no	[4]
32	not reported	TiO_2 with Au	Thermal oxidation (TiO_2) with electrodeposition (Au)	no	yes	no	no	[5]
9	not reported	$Ti_nM_mO_2$ (M: metal elements)	Laser molecular beam epitaxy	yes	no	no	no	[6]
63	not reported	$W_nO_mM_x$ (M: metal elements)	Electrochemical deposition	no	yes	no	no	[7]
63	not reported	ZnO with $EO_{20}PO_{70}EO_{20}$ as structure directing agent	Electrochemical deposition	no	yes	no	no	[8]
108	0.63 cm^2	$Zn_{1-x}Co_xO$	Electrochemical deposition	no	yes	no	no	[7]
42	$7.8 \cdot 10^{-3}$ cm^2	Fe-M-O and Bi-M-O (M: metal elements)	Metal organic decomposition	no	no	no	no	[9]
252	0.04 cm^2	Ternary metal oxides	Inkjet printing	no	yes	no	no	[10]
19600	$6.67 \cdot 10^{-4}$ cm^2	Multicomponent metal oxides	Inkjet printing	no	yes	no	no	[11]
64	6.25 cm^2	$Co_{3-x-y}Al_xFe_yO_4$	Inkjet printing	no	yes	no	no	[12]
1809	not reported	$Cu_2V_2O_7$ with different dopants	Inkjet printing	no	yes	no	no	[13]
5456	0.28 cm^2	(Fe-Co-Ni-Ti)O_x	Inkjet printing	no	yes	no	no	[14]
858	1 $\cdot 10^{-3}$ cm^2	$BiVO_4$ with Ni-La-Co-Ce catalysts	Spin coating ($BiVO_4$) and inkjet printing (catalysts)	no	yes	no	no	[15]
66	$1.6 \cdot 10^{-3}$ cm^2	$BiVO_4$ with different dopants	Drop casting	no	yes	no	no	[16]
20	$9 \cdot 10^{-4}$ cm^2	Fe_2O_3 with different dopants	Drop casting	no	yes	no	no	[17]
36	not reported	Fe_2O_3 with different dopants	Spray pyrolysis	yes	no	no	no	[18]
10	0.5 cm^2	Sn:Fe_2O_3	Spray pyrolysis	yes	yes	yes	yes	This work

Supplementary Note 1 - UV-visible spectroscopy and scanning electron microscopy

Reflectance and transmittance spectra of Fe_2O_3 photoelectrodes were measured with a Shimadzu UV-2600 UV-vis-NIR spectrophotometer with an integrating sphere (ISR-2600 PLUS Shimadzu). Absorbance spectra were then calculated and Tauc's equation was fitted supposing an allowed direct optical transition, obtaining the Tauc's plot of Figure S1. Fitting the linear part of the curve, in which parabolic band dispersion can be assumed, the optical bandgap of the Fe_2O_3 photoelectrodes is calculated to be 2.11 eV, which is coherent to the values of 1.9-2.2 eV reported in literature[19]. The morphology of the Sn:α-Fe_2O_3 thin films deposited on FTO was observed by scanning electron microscopy (Fig. S1). The film resulted fairly homogeneous and compact as in previous reports of α-Fe_2O_3 thin films obtained by spray pyrolysis[20,21]. The grain size of the film varied between 50 and 150 nm (Fig. S1 (a)) and the thin film thickness was approximately 100 nm (Fig. S1 (b)).

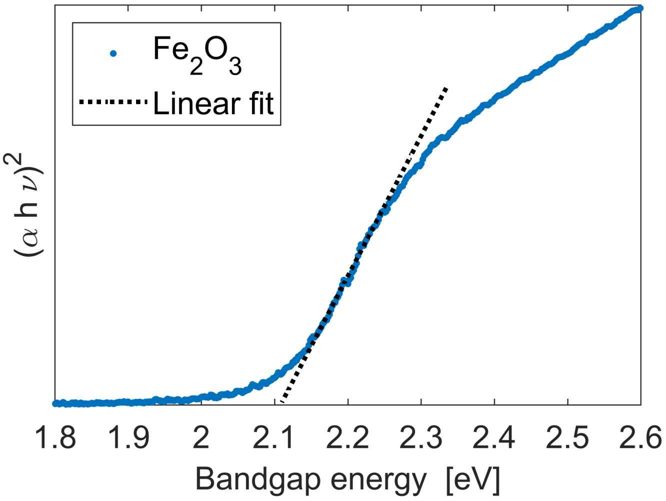

Figure S1: Tauc's plot of Fe_2O_3 photoanodes and linear fit of the region in which parabolic band dispersion can be assumed.

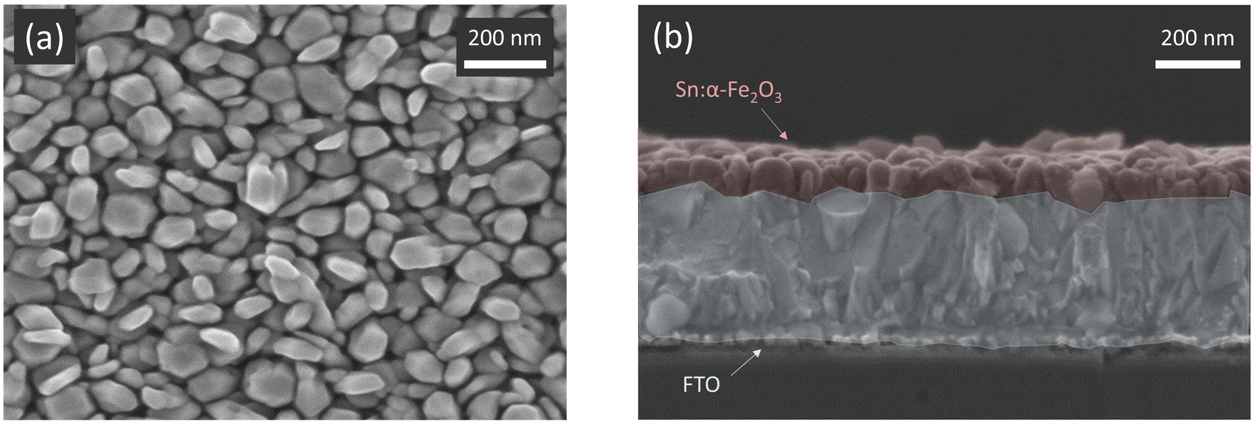

Figure S2: Scanning electron microscopy images of a Sn:α-Fe_2O_3 thin film on FTO after deposition by spray pyrolysis and annealing: (a) front view and (b) false-colour cross section view to highlight the two layers.

Supplementary Note 2 - LEDs characterization

Due to the need of operating ten light sources simultaneously, Light Emitting Diodes (LEDs) were chosen for their high efficiency, low cost and long lifespan. LEDs' emitted power was measured at different distances from the light sources through an Ocean Insight FLAME-S-XR1 spectrometer (optical resolution 1.7 nm FWHM) connected to an optical fiber (core diameter 300 μm, length 1 m) and a cosine corrector (CC-3-UV-S, OceanInsight), used to measure the hemispherical irradiance. The spectrometer was calibrated with a deuterium - tungsten halogen lamp (Ocean Insight DH-3P-BAL-CAL).

The spectral hemispherical irradiance at different distances from the light source of the ten LEDs used for the experimental campaign is reported in Fig. S3. The results of the characterization are summarized in Table S2.

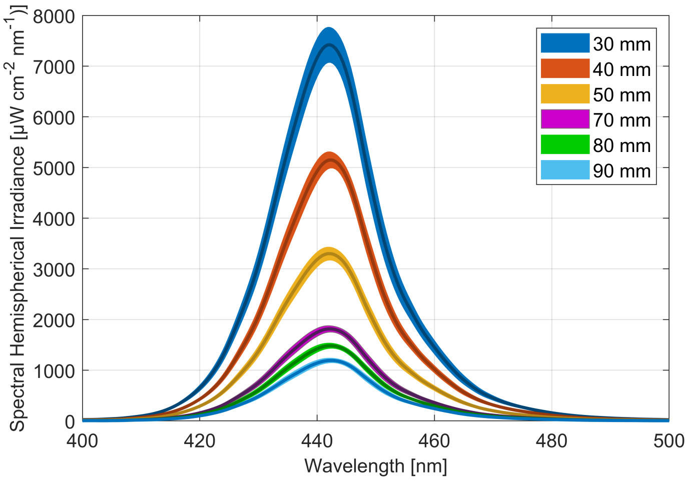

Figure S3: Average of ten LEDs spectral hemispherical irradiance measurements as a function of wavelength and error bars (in lighter colour) at distances from 30 to 90 mm from the light source.

The LEDs had the peak of spectral hemispherical irradiance at 442 nm, in the violet-blue region of visible spectrum, and a full width at half peak (FWHP) of 19 nm at every distance from the light source. The total hemispherical irradiance non-linearly decreased with distance.

The distance from the light source was chosen to simulate the irradiation from the global standard spectrum (AM 1.5G) despite the use of monochromatic light sources. To do so, we assumed: i) all the photons with energy lower than the energy bandgap of the semiconductor ($E < E_g$) are not absorbed by the photoelectrode; ii) for photons with energies larger or equal than the semiconductor energy bandgap ($E \geq E_g$), the only loss considered is the thermalization loss, i.e. the useful energy of the absorbed photon is E_g; iii) the blue LEDs used are ideal monochromatic light sources with characteristic wavelength $\lambda_{LED,c}$= 442 nm; iv) the function between two measured values of total hemispherical irradiance is linear; and v) the effect of reflection or diffraction of light due to the quartz window and the electrolyte are

Table S2: Wavelength of LEDs' spectral hemispherical irradiance peak, full width at half peak (FWHP) and total hemispherical irradiance at distances from 30 to 90 mm from the light source.

Distance from the LED [mm]	Wavelength of the peak [nm]	FWHP [nm]	Total hemispherical irradiance [W m^{-2}]
30	442	19	1769 ± 198
40	442	19	1221 ± 67
50	442	19	787 ± 71
70	442	19	433 ± 36
80	442	19	357 ± 25
90	442	19	289 ± 27

not considered. They imply:

$$G_{AM1.5G,useful} \simeq \int_0^{\lambda_g} G_{\lambda,AM1.5G} \frac{\lambda}{\lambda_g} d\lambda \simeq G_{LED,useful} \simeq G_{LED} \frac{\lambda_{LED,c}}{\lambda_g} \tag{1}$$

$$G_{LED} = \frac{\lambda_g}{\lambda_{LED,c}} \int_0^{\lambda_g} G_{\lambda,AM1.5G} \frac{\lambda}{\lambda_g} d\lambda \tag{2}$$

$G_{AM1.5G,useful}$ is the total irradiance from the AM 1.5G spectrum which contributes, under the aforementioned assumptions, to a useful effect with the Fe_2O_3 photoanodes [W m^{-2}]; $G_{\lambda,AM1.5G}$ is the spectral irradiance from the AM 1.5G spectrum [W m^{-2} nm^{-1}], obtained from the PV Lighthouse database[22]; $G_{LED,useful}$ is the total irradiance from the blue LEDs which contributes to a useful effect with the Fe_2O_3 photoanodes [W m^{-2}]; G_{LED} is the total irradiance from the blue LED [W m^{-2}], λ is photons wavelength [nm]; λ_g= 590 nm is the wavelength of the photon with energy equal to the semiconductor energy bandgap (E_g = 2.1 eV).

Under these assumptions, the calculated irradiance from the LED is G_{LED} = 348 W m^{-2} and it is obtained at a distance from the light source of 81 mm, as shown in Figure S4.

To prove that this approach is consistent, the number of photons reaching the photoelectrode with AM 1.5G spectrum and with the blue LEDs are compared. The energy of the photons emitted by the LEDs is 2.804 eV, *i.e.* its wavelength is $\lambda_{LED,c} = 442$ nm. With an irradiance of 348 W m^{-2}, the flux of photons $\Phi_{LED} = 7.75 \cdot 10^{20} m^{-2} s^{-1}$, which is 0.9% more than the cumulative photon flux with energies higher than the semiconductor energy bandgap from AM 1.5G spectrum, $\Phi_{AM1.5G,E>E_g} = 7.68 \cdot 10^{20} m^{-2} s^{-1}$. This confirms that it is a good assumption to place the photoelectrode at a distance of 81 mm from the LED light source to simulate the AM 1.5G spectrum.

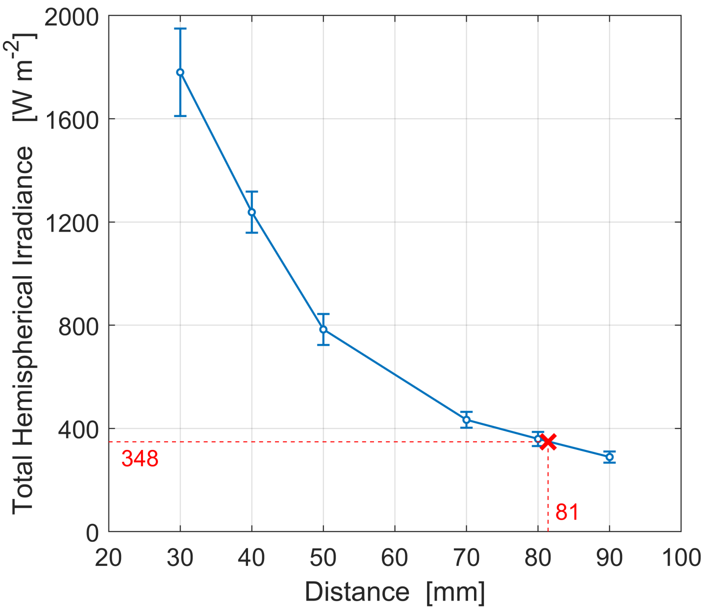

Figure S4: Total hemispherical irradiance of the LEDs as a function of the distance from the light source and (in red) graphical method to identify the distance from the light source for which the irradiance corresponds to the one of the AM 1.5G spectrum.

Supplementary methods

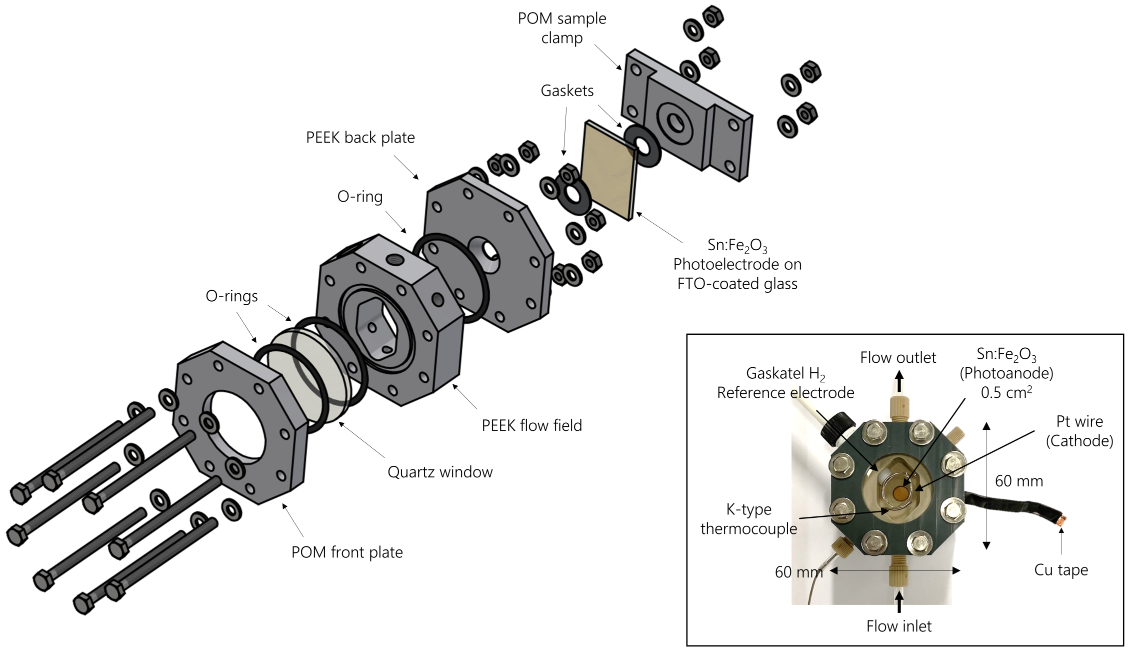

Figure S5: Exploded view and front view (in the inset) of the PEC cell used for the parallel photoelectrochemical tests.

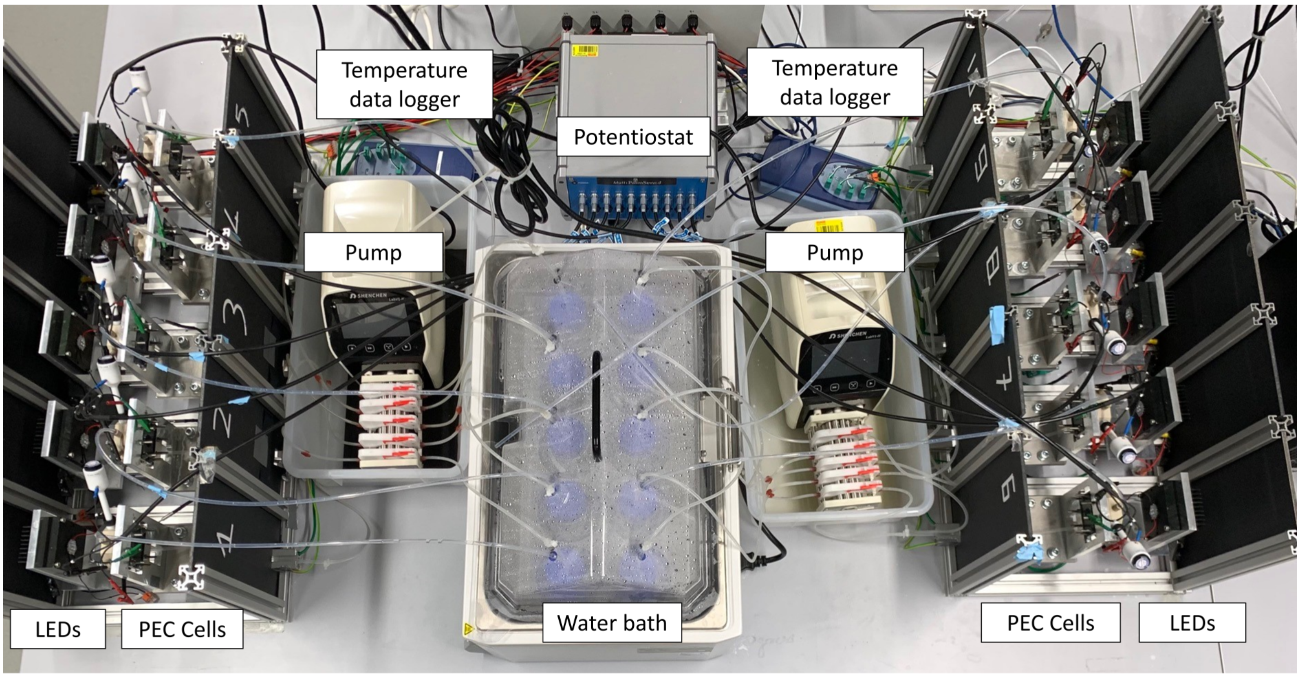

Figure S6: Bird-view picture of the setup to test ten PEC cells in parallel.

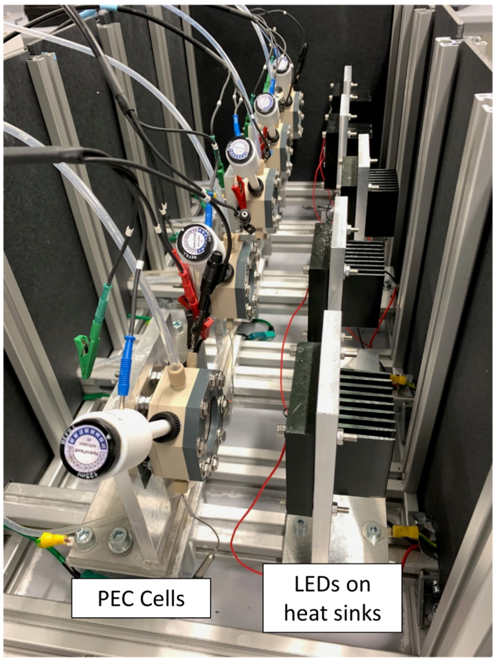

Figure S7: Detail of one of the two sides of the setup with five PEC cells in front of five LEDs, each mounted on a heat sink.

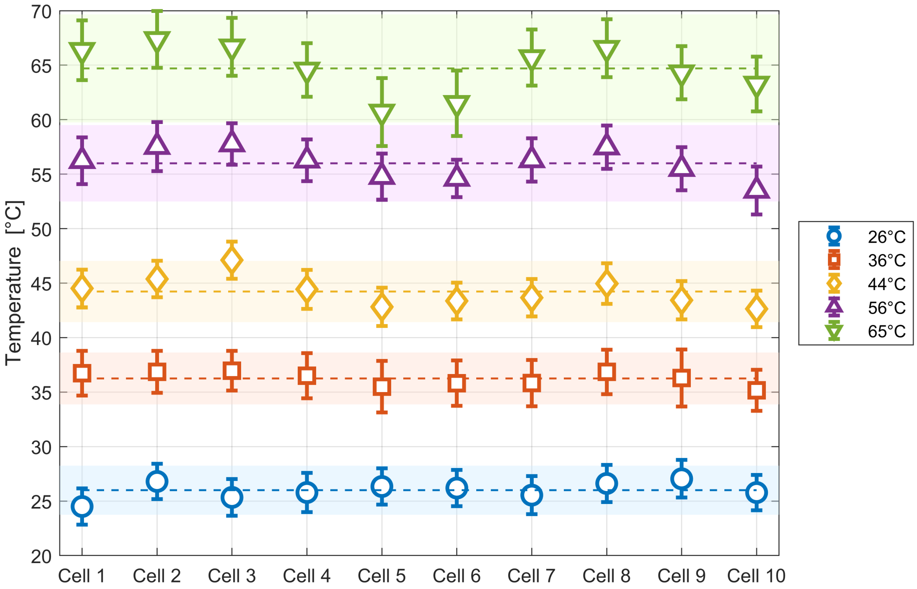

Figure S8: Temperature distribution in the ten PEC cells during the photoelectrochemical tests. Contrarily to the rest of the manuscript, the error bars are obtained from the temporal standard deviation of the single measurements, the dashed lines are the average temperatures across the ten cells and the colourbands are their error bars obtained from the spatial standard deviations of the ten measurements.

Supplementary results

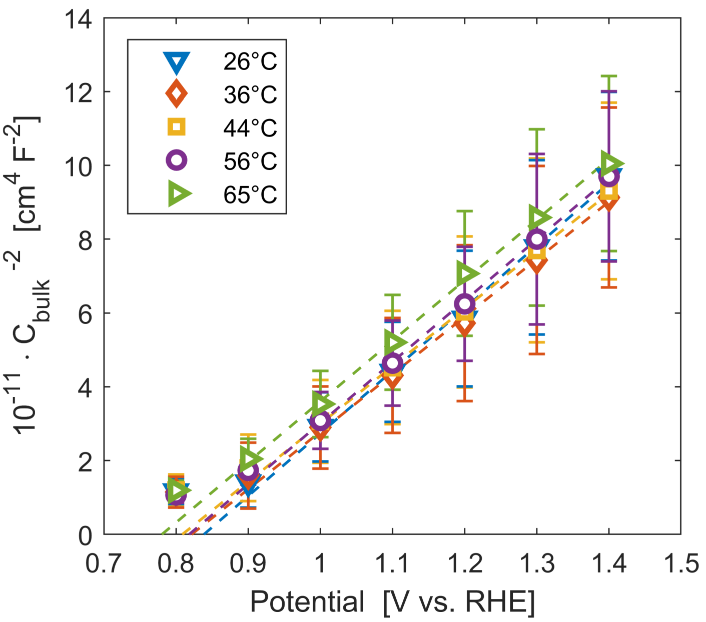

Figure S9: Mott-Schottky plots of Sn:α-Fe_2O_3 thin films at different temperatures in dark and linear regression of the linear region of the curve (dashed lines).

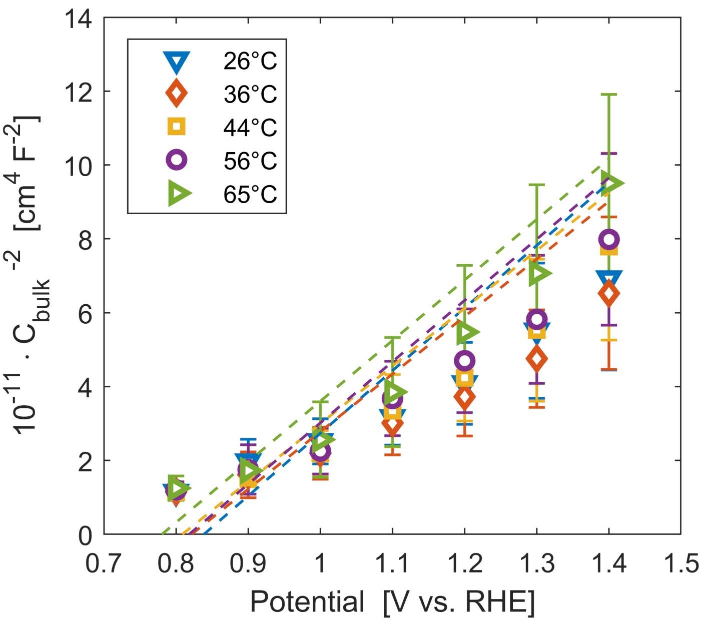

Figure S10: Mott-Schottky plot in blue light at different temperatures compared to the linear fit of the Mott-Schottky plot in dark (dashed lines) to underline the effect of surface state pinning.

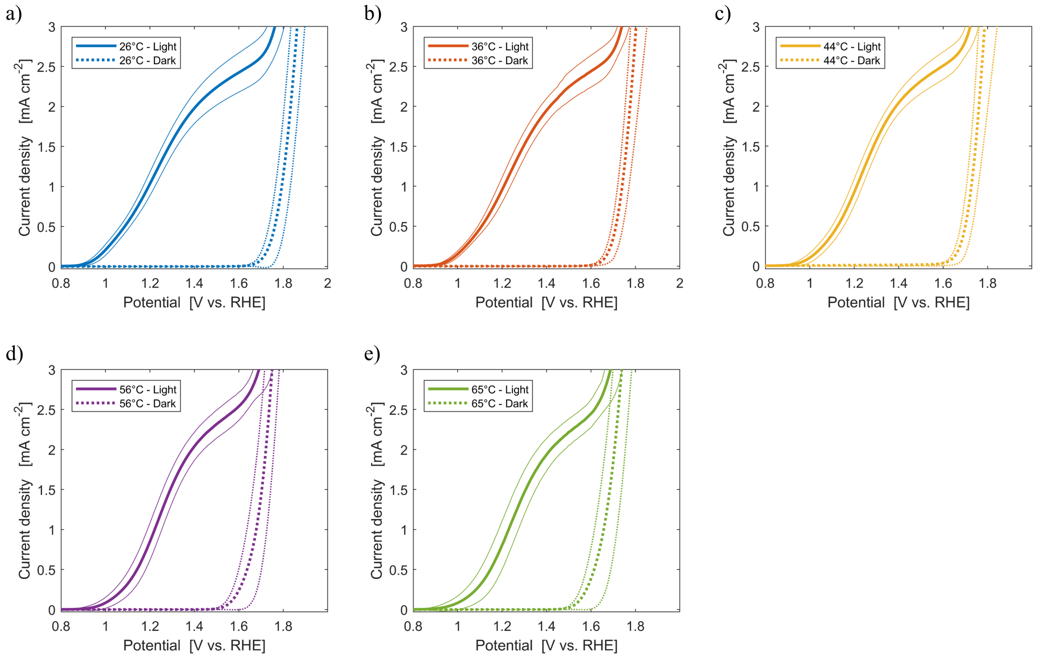

Figure S11: Current density-applied potential characteristic curves with error bars obtained averaging ten parallel cyclic voltammetries forward sweeps of Sn:Fe_2O_3 thin films in blue light (solid line) or dark (dotted line) at (a) 26°C, (b) 36°C, (c) 44°C, (d) 56°C, (e) 65°C.

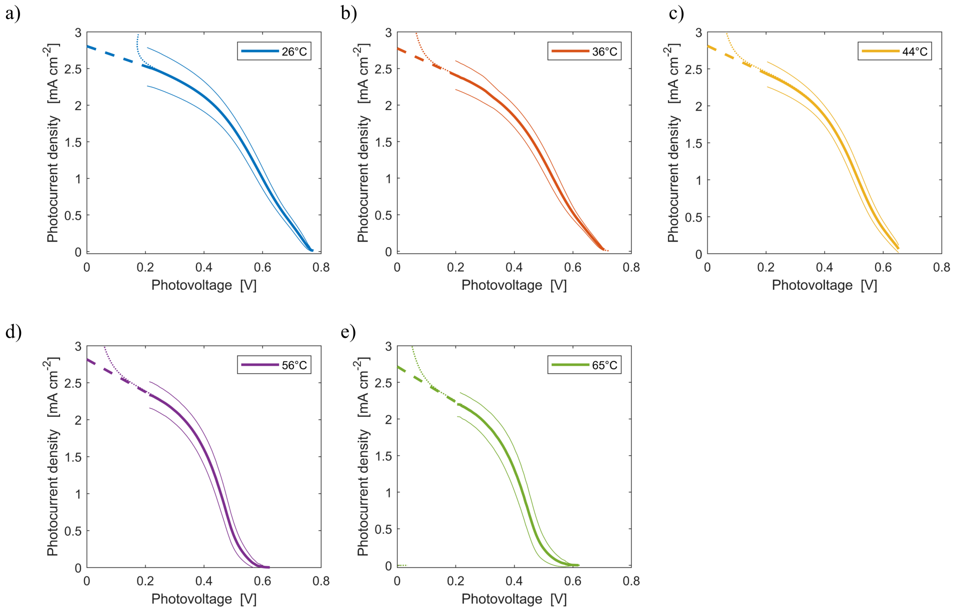

Figure S12: Photocurrent density-photovoltage characteristic curves with error bars obtained from ten parallel measurements with Sn:Fe_2O_3 thin films in blue light (solid line) at (a) 26°C, (b) 36°C, (c) 44°C, (d) 56°C, (e) 65°C. Dotted lines represent the measured photocurrent density at low photovoltages and dashed lines the photocurrent densities extracted linearly fitting the saturation regions of the curves.

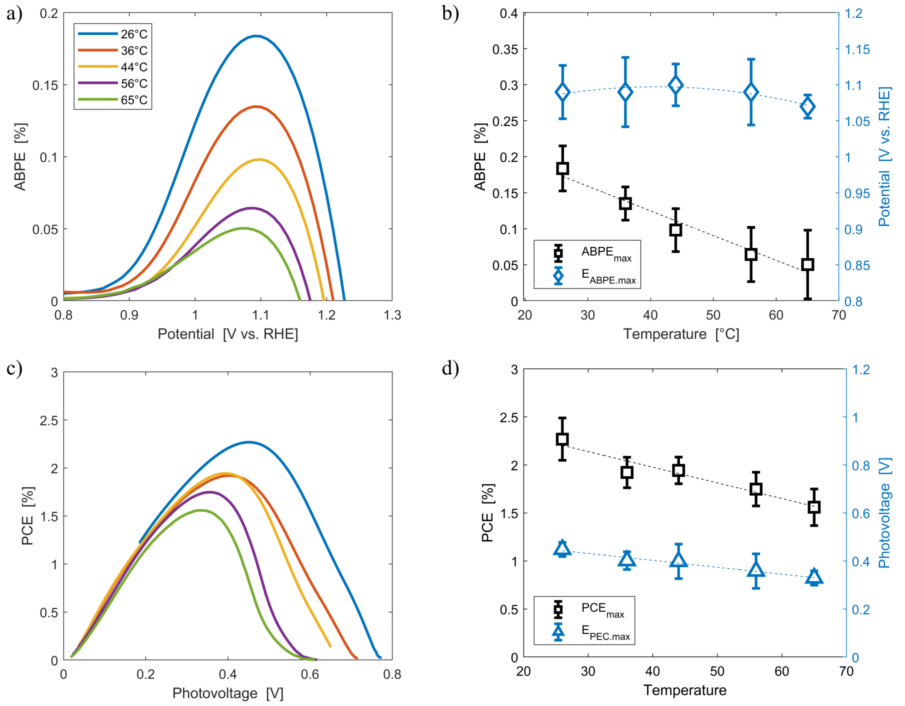

Figure S13: (a) Sn:α-Fe_2O_3 thin films applied bias photon-to-current efficiency (ABPE) as a function of applied potential at different temperatures. (b) Maximum ABPE (left axis) and potential at which the maximum ABPE is measured (right axis) as a function of temperature. (c) Sn:α-Fe_2O_3 thin films apparent photon-to-current (PCE) efficiency as a function of photovoltage at different temperatures. (d) Maximum apparent PCE (left axis) and photovoltage at which the maximum apparent PCE is measured (right axis) as a function of temperature. In (b)-(d) the dashed lines are the regressions of the experimental data.

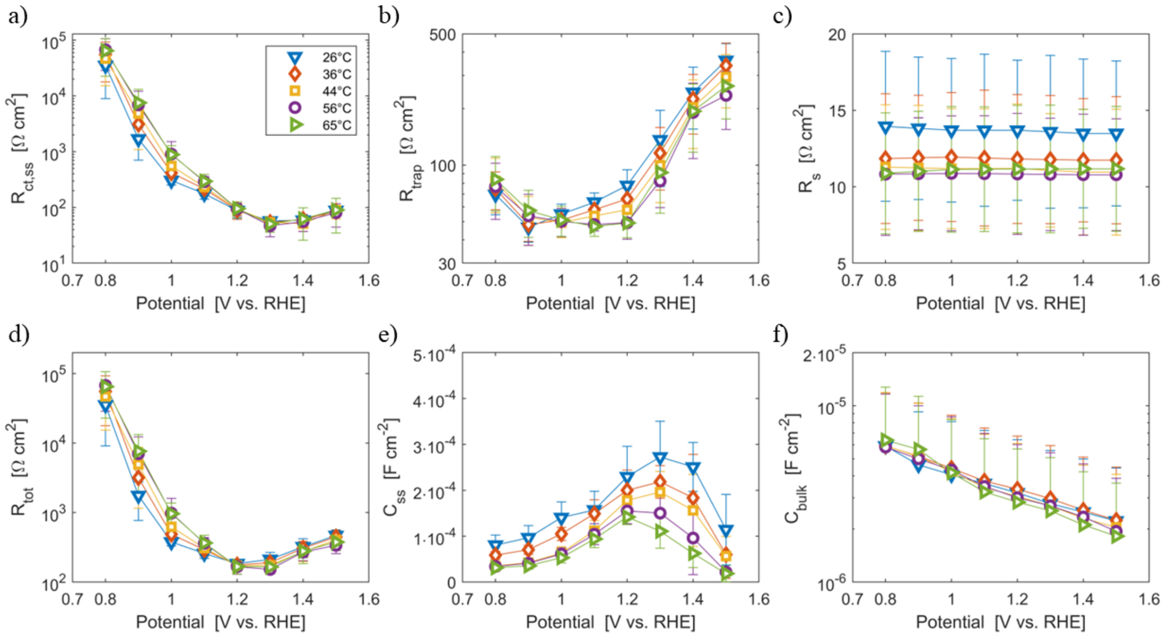

Figure S14: Resistances and capacitances extracted fitting the electrochemical impedance spectroscopy curves at direct potentials from 0.8 to 1.5 V vs. RHE in blue light with the equivalent circuit of Figure 1 (b) .

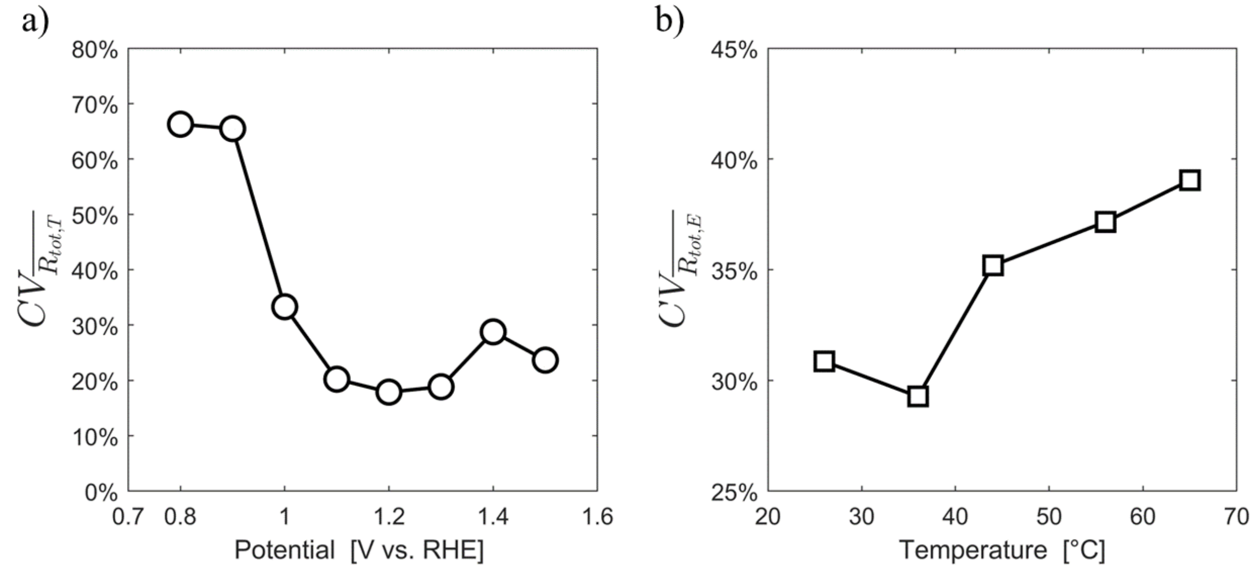

Figure S15: Coefficient of variance of the total resistance $R_{\rm tot}$ (a) averaged in temperature as a function of applied potential and (b) averaged in applied potential as a function of temperature.

References

[1] K. Sliozberg, D. Schäfer, T. Erichsen, R. Meyer, C. Khare, A. Ludwig and W. Schuhmann, *ChemSusChem*, 2015, **8**, 1270–1278.

[2] K. J. Jenewein, S. Thienhaus, A. Kormányos, A. Ludwig and S. Cherevko, *Chemical Science*, 2022, **13**, 13774–13781.

[3] L. Zhou, Y. Wang, K. Kan, D. M. Lucana, D. Guevarra, Y. Lai and J. M. Gregoire, *ACS Sustainable Chemistry & Engineering*, 2022, **10**, 15898–15908.

[4] Z. Zhang, S. A. Lindley, D. Guevarra, K. Kan, A. Shinde, J. M. Gregoire, W. Han, E. Xie, J. A. Haber and J. K. Cooper, *Advanced Functional Materials*, 2020, **30**, 2000948.

[5] S.-H. Baeck, T. F. Jaramillo, A. Kleiman-Shwarsctein and E. W. McFarland, *Measurement Science and Technology*, 2005, **16**, 54–59.

[6] A. Nakayama, E. Suzuki and T. Ohmori, *Applied Surface Science*, 2002, **189**, 260–264.

[7] T. F. Jaramillo, S.-H. Baeck, A. Kleiman-Shwarsctein, K.-S. Choi, G. D. Stucky and E. W. McFarland, *Journal of Combinatorial Chemistry*, 2005, **7**, 264–271.

[8] T. F. Jaramillo, S.-H. Baeck, A. Kleiman-Shwarsctein and E. W. McFarland, *Macromolecular Rapid Communications*, 2004, **25**, 297–301.

[9] T. Arai, Y. Konishi, Y. Iwasaki, H. Sugihara and K. Sayama, *Journal of Combinatorial Chemistry*, 2007, **9**, 574–581.

[10] J. E. Katz, T. R. Gingrich, E. A. Santori and N. S. Lewis, *Energy & Environmental Science*, 2009, **2**, 103–112.

[11] M. Woodhouse, G. S. Herman and B. A. Parkinson, *Chemistry of Materials*, 2005, **17**, 4318–4324.

[12] M. Woodhouse and B. A. Parkinson, *Chemistry of Materials*, 2008, **20**, 2495–2502.

[13] P. F. Newhouse, D. Guevarra, L. Zhou, Y. Wang, M. Umehara, D. A. Boyd, J. M. Gregoire and J. A. Haber, *Matter*, 2020, **3**, 1601–1613.

[14] J. M. Gregoire, C. Xiang, X. Liu, M. Marcin and J. Jin, *Review of Scientific Instruments*, 2013, **84**, 024102.

[15] D. Guevarra, A. Shinde, S. K. Suram, I. D. Sharp, F. M. Toma, J. A. Haber and J. M. Gregoire, *Energy & Environmental Science*, 2016, **9**, 565–580.

[16] H. Ye, J. Lee, J. S. Jang and A. J. Bard, *The Journal of Physical Chemistry C*, 2010, **114**, 13322–13328.

[17] J. S. Jang, J. Lee, H. Ye, F.-R. F. Fan and A. J. Bard, *The Journal of Physical Chemistry C*, 2009, **113**, 6719–6724.

[18] A. Kleiman-Shwarsctein, P. Zhang, Y. Hu and E. W. McFarland, *On Solar Hydrogen & Nanotechnology*, John Wiley & Sons, Ltd, 2010, pp. 401–458.

[19] T. Lindgren, L. Vayssieres, H. Wang and S.-E. Lindquist, *Chemical physics of nanostructured semiconductors*, 2003, 83–110.

[20] P. Dias, A. Vilanova, T. Lopes, L. Andrade and A. Mendes, *Nano Energy*, 2016, **23**, 70–79.

[21] C. Jorand Sartoretti, B. D. Alexander, R. Solarska, I. A. Rutkowska, J. Augustynski and R. Cerny, *The Journal of Physical Chemistry B*, 2005, **109**, 13685–13692.

[22] *PV Lighthouse AM1.5G spectrum*, https://www2.pvlighthouse.com.au/resources/optics/spectrum.
